# IndoorCare: Low-Cost Elderly Activity Monitoring System through Image Processing

**DOI:** 10.3390/s21186051

**Published:** 2021-09-09

**Authors:** Daniel Fuentes, Luís Correia, Nuno Costa, Arsénio Reis, José Ribeiro, Carlos Rabadão, João Barroso, António Pereira

**Affiliations:** 1 Computer Science and Communication Research Centre, School of Technology and Management, Polytechnic Institute of Leiria, 2411-901 Leiria, Portugal; daniel.fuentes@ipleiria.pt (D.F.); luis.correia@ipleiria.pt (L.C.); nuno.costa@ipleiria.pt (N.C.); jose.ribeiro@ipleiria.pt (J.R.); carlos.rabadao@ipleiria.pt (C.R.); 2 INESC TEC, University of Trás-os-Montes e Alto Douro, Quinta de Prados, 5001-801 Vila Real, Portugal; ars@utad.pt (A.R.); jbarroso@utad.pt (J.B.); 3 INOV INESC Inovação, Institute of New Technologies, Leiria Office, Campus 2, Morro do Lena-Alto do Vieiro, Apartado 4163, 2411-901 Leiria, Portugal

**Keywords:** computer vision, image analysis, internet of things, monitoring of elderly, low cost

## Abstract

The Portuguese population is aging at an increasing rate, which introduces new problems, particularly in rural areas, where the population is small and widely spread throughout the territory. These people, mostly elderly, have low income and are often isolated and socially excluded. This work researches and proposes an affordable Ambient Assisted Living (AAL)-based solution to monitor the activities of elderly individuals, inside their homes, in a pervasive and non-intrusive way, while preserving their privacy. The solution uses a set of low-cost IoT sensor devices, computer vision algorithms and reasoning rules, to acquire data and recognize the activities performed by a subject inside a home. A conceptual architecture and a functional prototype were developed, the prototype being successfully tested in an environment similar to a real case scenario. The system and the underlying concept can be used as a building block for remote and distributed elderly care services, in which the elderly live autonomously in their homes, but have the attention of a caregiver when needed.

## 1. Introduction and Motivation

Portugal has an aging population with a tendency to increase [1], particularly, in rural areas, where the migration of the active population to large urban centers, in search of better job opportunities and quality of life, has led the remaining resident population in rural environments, mostly elderly, suffering social exclusion, and often ending up living in isolation.

As the economic factor is sometimes a barrier for technology adoption, especially by the elderly population with limited financial resources [2], cost-effective solutions to monitor the elderly and the isolated population in general have been researched for a while. The Ambient Assisted Living Joint Programme [3] is a European initiative and is an example of the needs that exist in support for the elderly. Since then, more and more research has been carried out [4], and every year new solutions appear make their contribution.

In this article, a low-cost solution for monitoring the movement of elderly people living alone in their homes, based on the AAL paradigm, is presented with the name IndoorCare. The system is based on a distributed architecture, where low-cost IoT devices acquire and process video images to export non-personal/private data through a gateway to a server. Then, the server aggregates all this information and makes it available, in a simple way, to the user or caregiver. This proposed solution is based in technologies increasingly used in the area of smart everything [5] and provides a non-invasive monitoring system to a caregiver or a family member. The IndoorCare system records the person’s physical movements over time and allows that information to be analyzed later by the caregiver to assess the person’s health. One other advantage of a solution such as this is to allow the detection of any anomalous situations, such as emergencies or possible falls, in time.

The paper is organized as follows: Section 2 presents an overview of the related work; Section 3 describes the solution’s architecture; Section 4 presents the prototype that was developed to validate the concept; Section 5 presents the system’s evaluation and optimizations; and Section 6 presents the work’s general conclusions.

## 2. Related Work

There are several solutions and projects focused on the detection, spatial location, and monitoring of the daily activity of people in indoor environments. In this section, we discuss some of the founding technologies for this type of solution, namely image analysis and infrared sensors.

There are several articles that analyze the AAL solutions that have been appearing in recent years [6,7] and some that expose the challenges that must be resolved in the future [8], especially in a post COVID-19 era [9]. There are also recent studies that focus on the analyses and comparison, in various ways, of the created applications and architectures of recent AAL solutions, exposing the trends in the solutions’ implementation that most works have followed [10]. On a more practical level, there are several interesting implementations that have used various methods to monitor and interact with older people; these solutions typically use IoT devices to perform this monitoring [11], whether using sensors attached to the person [12], monitoring furniture [13], using video systems that analyze in real time what is happening [14,15], using face recognition to detect people and who they are [16], or even through the analyses of the sound [17]. The use of computer vision together with artificial intelligence is an increasingly common practice, using the best that these technologies allow to better monitor the elderly in their homes [18]. In addition, there is a growing need to transfer the information processing from data centers to the periphery of the systems, namely to the source where the data is acquired, to reduce the traffic sent by the equipment at the edge. This concept is called fog computing, and in addition to being a great advantage for computer vision solutions, it is also already being used in monitoring solutions for the elderly that use wearable devices, among others [19].

The extraction of information based on image analysis is a relatively recent topic that has enabled the development of technologies that allow the automatization of the information gathering process. Image recognition solutions, such as the Open-Source Computer Vision Library (OpenCV) [20], combined with ubiquitous computing, using microcomputers, such as Raspberry Pi [21] or Arduino [22], allow the creation of environments that can act intelligently, according to the information extracted and collected.

The OpenCV software library uses image analysis to recognize the various types of information in an image, such as: detection of hand gestures, as set out in [23]; human facial recognition [24], where in this particular paper [25] the authors implemented a prototype using the Java CV library [26], which analyzes the camera stream from a IP security camera and detects human presence; and recognition and extraction of vehicle registration information [27] or surveillance security systems [28], in which the authors coupled common web cameras to devices of small processing power, e.g., Raspberry Pi, which acquires the image from the camera and uses a cloud platform to process the image for movement detection.

The research in the area of human monitoring and human location has resulted in several interesting works, such as the one presented in [29], where the authors propose a system that uses two modes of monitoring, inside the residence (indoor) and outside the residence (outdoor). For the detection of the indoor position, the users must wear RFID (Radio-Frequency Identification) tags, which are detected and read whenever the user enters a new division, similarly to the RFID tagging systems used in logistic solutions to track items. For the detection of the outdoor position, the user must wear a GPS (Global Positioning System) device for position tracking. The GPS mode (outdoor) is activated automatically whenever the user leaves the room three meters away.

In ref. [30], the authors used infrared (IR) sensors to calculate the number of people inside a building, installing sensors on the doors’ tops to detect transit movement between rooms, so they could calculate how many people were in each division.

In the work developed in ref. [31], the authors propose a solution that addresses some of the problems enunciated in this work. A system is proposed that constantly monitors the security of a home. It uses several Raspberry Pi devices, connected to surveillance cameras, and uses the OpenCV library for image analysis. The system can detect various types of events, such as opening and closing doors and windows, movement in the rooms, and breaking windows.

Table 1 summarizes and explains why the solutions previously presented are considered interesting for the development of this solution.

The work that identifies most of the requirements for this intended solution is ref. [31], mainly because of the image analysis using computer vision, artificial intelligence, and IoT devices. Although the solution works as intended, according to the authors, it requires too many processing resources from the IoT devices, which means that a robust IoT device must be used, thus increasing the solution’s cost. The objective of this work is to develop a solution that can monitor a person’s movements inside the house, in the various rooms, also using computer vision, as in ref. [31], using IoT devices, while keeping the cost reasonably low.

## 3. IndoorCare System Architecture

**Figure 1:**
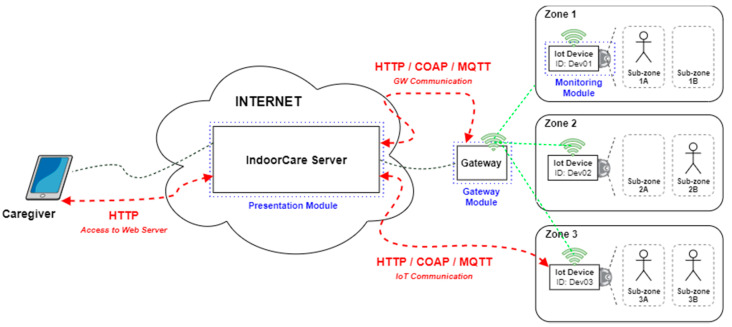
IndoorCare conceptual architecture.

The solution IndoorCare, proposed in this article, is based on some principles used in other solutions, namely using only one device per room for human detection [29], detecting the presence of people through motion capture analysis [30], and using low-cost microcomputers to analyze and process the collected information [31]. The system has a distributed and multi-agent architecture [32], which implements the client–server model, having a gateway module to ensure information security, as presented in Figure 1.

The architecture comprises three modules, highlighted in Figure 1, where each module has a specific agent, a software-based entity, which performs the various tasks on the equipment to ensure proper operation of the system:

- The monitoring module, which translates to the several IoT devices at home that capture images and pre-process the data obtained from the images, in order to send this information to the presentation module.
- The gateway module, responsible for ensuring WiFi network availability and data communication security from the clients to the server (presentation module).
- The presentation module, which is the server that receives the data from the monitoring modules. It provides a presentation layer for the users (caregivers) to visually perceive the dynamics of the elderly person’s activities inside the house over time.

The option to use image acquisition equipment and computer vision, instead of infrared sensors, although the latter in theory are cheaper, was because with computer vision it is possible to analyze several subzones within the same zone with a single IoT device, while with affordable infrared sensors, typically, one sensor is necessary to detect motion in each subzone. Although there are infrared sensors with this capability, such as the temperature detection cameras used to detect possible cases of COVID-19 [33], these are not low-cost IoT devices as their cost is in the range of thousands of euros per device.

**Figure 2:**
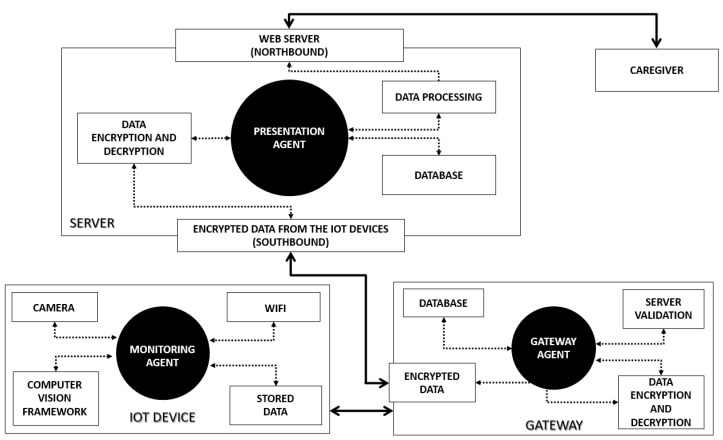
IndoorCare detailed architecture.

In Figure 2 are detailed all the components of each of the modules and how they communicate with each other.

### 3.1. Monitoring Module (IoT Device)

The IoT devices present in the elderly person’s home are responsible for processing the information they acquire; namely, image analysis through OpenCV, generating processed data ready to be sent to the server. This functionality is in line with the Edge Computing concept [34], where the processing is executed close to the data source, in this case at the home of the elderly, being a paradigm increasingly used in the IoT universe and in smart systems.

The monitoring module works as a black box system that receives video feeds, and outputs the extracted data from the image analysis in the form of movement events. No other information is fed to the user/caregiver. This module encompasses the devices installed in the distinct areas (zones) of the elderly home to perform the movement detection. The software agent defined for this module and presented in Figure 2 is responsible for the image acquisition and hotspot calculation, using the available resources on the IoT device. A hotspot is defined as an area where movement is detected in the image, and for which the device must compare several image frames to be able to confirm if there is movement in a zone or not.

Each equipment has a unique identifier (ID) that identifies with which zone the device is associated. The ID is also used to identify the hotspot’s information in the server’s database and establish a relation between the device and its location in the house.

### 3.2. Gateway Module (Gateway)

This module is responsible for the confidentiality and integrity of the data transmission between the monitoring module and the presentation module. The module’s agent, also presented in Figure 2, ensures that all communications go through an encrypted tunnel, from the network access point to the server. The agent guarantees the data encryption, as well as the server’s address validation.

### 3.3. Presentation Module (Server)

This module acts as the server element of the client–server model in the communication and receives data from the clients, which are represented by the monitoring module. The data are saved, processed, and presented to the caregiver user. The software agent in this module, as presented in Figure 2, implements the features for data reception, decryption, saving, and presentation on a web platform. The user interaction is minimalistic and the agent basically combines the data streams from the several clients into a unique event feed.

To create the event stream from the data, the agent uses the subzones, as previously defined in the server’s configuration, to check whether there is movement within them, using the hotspots sent by the clients. A subzone corresponds to a part of the image (the entire zone) acquired by a particular IoT device and corresponds to a specific area inside the elderly person’s home.

This module provides a web portal for the caregiver to monitor the elderly and browse the daily activities inside the house, via Northbound [35] access. It also provides communication using Application Programming Interfaces (API) [36] via Southbound [35] for communication with the IoT devices and gateways.

### 3.4. Communication

As seen previously in Figure 1, there are three different types of communications:

- Caregiver with the server;
- Gateway with the server;
- IoT devices with the server.

In Northbound communication, between the caregiver and the server, as it typically occurs in a web environment (via the Internet), the most suitable communication protocol will be HTTP (Hypertext Transfer Protocol), in its secure version (HTTPS) [37]. This is one of the most used protocols for accessing online platforms and is widely used in the IoT environment for the same purpose.

In Southbound communication, between the server, gateways, and IoT devices, since it is a communication between IoT and network devices (if supported by the hardware), several protocols focused on the IoT environment can be used:

- HTTP (Hypertext Transfer Protocol): The most used client–server communication protocol on the Web which is also widely used in the IoT world due to its simplicity and efficiency in delivering information.
- COAP (Constrained Application Protocol): A communication protocol designed for devices that have limited processing capabilities, much like HTTP, but that uses much less data to send messages.
- MQTT (Message Queuing Telemetry Transport): One of the lightest communication protocols, it uses the Publisher/Subscriber model to exchange messages and is widely used in scenarios where network connectivity is not ideal.

These are just a few examples of communication protocols that can be implemented in this architecture, with HTTP still being one of the most used [38].

## 4. Implemented Prototype

In this section is presented the prototype developed to validate the proposed architecture. Low-cost IoT devices, widely used by the community, were used to implement the solution to validate the fulfilment of the objectives set out in the previous section.

**Figure 3:**
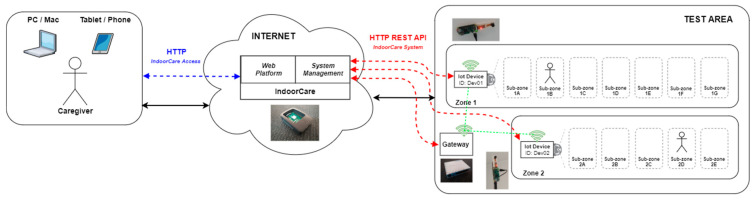
Prototype conceptual architecture.

In Figure 3, the general architecture of the prototype is illustrated, showing the modules and devices used.

The implemented prototype incorporates all the modules described in the architecture to demonstrate the intended functionality with the proposed system. Links at the right side are the interaction that takes place between the server, gateways and IoT devices (Southbound). The link at the left side represents the user/caregiver interaction with the web platform to access the IndoorCare system (Northbound). It should be noted that in this prototype, at the gateway level, only the basic static mechanisms were implemented for the system to work correctly, namely the VPN connection and server address validation.

### 4.1. Equipment Used

The equipment selection for this prototype project considered the costs in order to keep the solution effective and as low cost as possible, targeted at people with modest economic resources.

**Figure 4:**
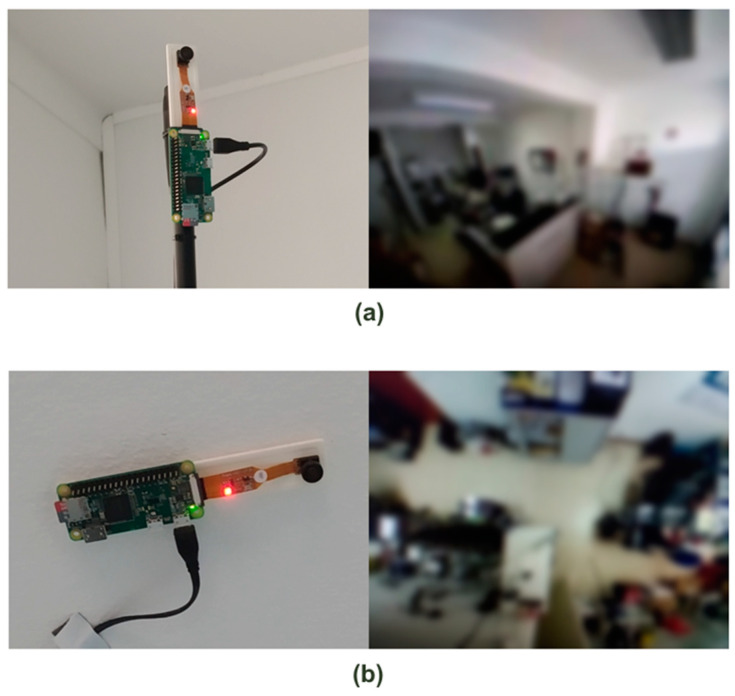
Raspberry PI Zero W #1 (**a**) and #2 (**b**).

For the client IoT devices, we opted to use Single Board Computers (SBC) with an Operating System (OS) based in Linux, specifically the from the Raspberry PI family [39], due to its low price and good technical characteristics. To capture and analyze the images, we chose the Raspberry Pi Zero W [40] combined with a Fisheye 160° camera (including a 5 V 2.1 A power supply and Micro SD card), as presented in Figure 4. The total cost per device was around EUR 45 + VAT.

This equipment is reasonably compact and has a set of ideal characteristics, such as a single-core processor at 1 GHz, 512 MB of RAM, and built-in WiFi, thus enabling the creation of client equipment capable of collecting, processing, and sending images to the system server over a WiFi network. It should be noted that initially the cameras used were normal Raspberry Pi Camera Modules [41], which were replaced by fisheye cameras only after testing the system, as described in Section 5.

For the server equipment, to receive, store, process, and present data on a web portal, we opted for the Raspberry Pi 3 B [42] (including a 5 V 2.1 A power supply and a Micro SD card). The total cost was around EUR 50 + VAT. The characteristics of this equipment, despite being an IoT device, meet the requirements for server equipment, as it has a quad-core processor at 1.4 GHz and 1 GB of RAM. Although this device has a reasonable performance, in a real or production environment, a more robust computational node should be used, namely a dedicated server (PC) or an online VPS (Virtual Private Server).

For network gateway equipment, we chose a Mikrotik Routerboard RB951Ui-2ND [43], mainly because of the possibility to create internal scripting for network management, and the ability of this scripting to communicate with platforms via REST API. This device had a total cost around EUR 30 + VAT. This equipment can work as a WiFi access point for the client devices, allowing the creation of a secure bridge Virtual Private Network (VPN) [44] between client and server equipment, thus ensuring client–server end-to-end confidentiality.

**Figure 5:**
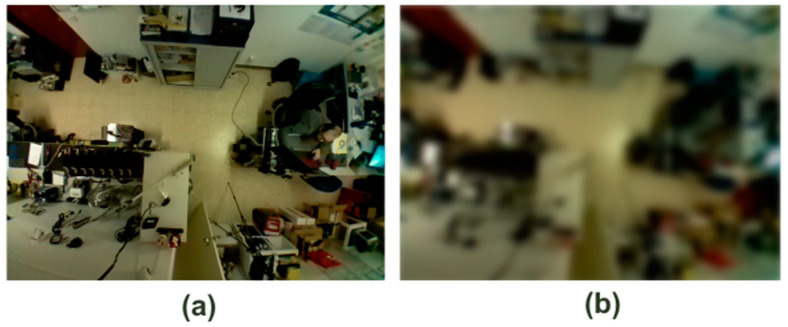
Focused (**a**) and unfocused (**b**) lens.

One major concern is the device intrusion that can lead to the visualization of the images captured by the camera by unauthorized persons. A way to guarantee the privacy of the residents is by physically blurring the lens of the equipment. Figure 5 shows the differences between a focused and an unfocused lens, and it is possible to notice that in the image with the lens out of focus, objects and people are not perceptible, thus ensuring the privacy required by the GDPR [45].

### 4.2. System Operation

To acquire the location of an individual person from video camera images, it is necessary to analyze and extract information from the images. We used the OpenCV software library [20] and the ImUtils library [46] to recognize movement in the images and Python [47] as the programming language for the software agent. It is possible to compare two images, one that serves as base reference for comparison and the other to check for changes, converting the captured images to arrays of pixels and comparing the different values of their respective positions. To perform the comparison, the absolute value in the subtraction of each of the respective pixels is obtained, thus creating an image that presents the differences found in the pixel array, which in this case shows the complete changes that occurred between the images. Then, a threshold is applied to the resultant image, by defining a change limit between pixels, where the pixels below the threshold are discarded and those above are saved, to create an image, commonly known as threshold, which contains only the pixels where there is a significant difference or, in this case, movement detection.

**Figure 6:**
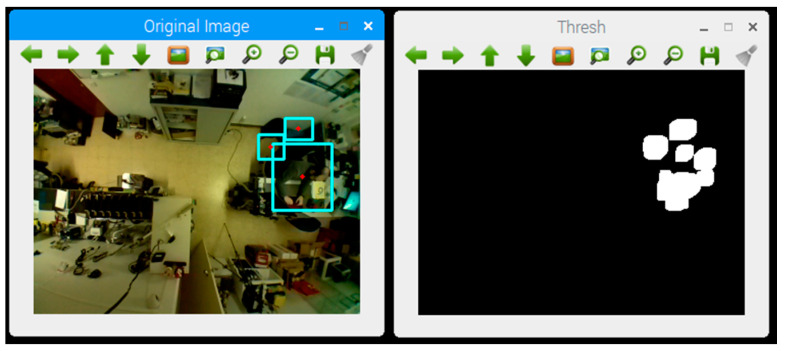
Motion detection in the image.

In Figure 6, on the right side, the threshold which corresponds to the movement detected on the left side of the image is displayed, with the movement area defined by a blue rectangle.

**Figure 7:**
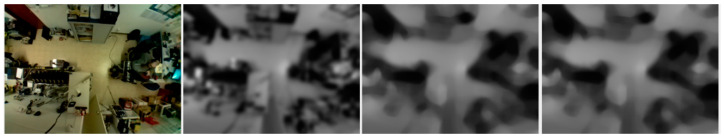
Some types of image blurring.

To effectively calculate the threshold, as stated in [24], it is necessary at an early stage to convert the color image to a grayscale image, so the only differentiating factor is the pixel brightness. Then, it is necessary to blur the image so that there are no sudden changes in the pixel tones. Figure 7 shows the different types of blurs supported by OpenCV: Gaussian Blur, Median Blur, and Normalized Block Blur [48].

Each blur type uses a different approach, yielding different results. The tests performed consisted of acquiring images where there was always the same human movement, walking from one end of the room to the other. Several threshold values were tested with the different types of blurs, which led to the following conclusion:

- Median Blur and Normalized Block give less false positives in the motion detection;
- Gaussian Blur detects more movement, as it provides more image detail after blurring.

In the prototype, Median Blur was used, but any other blur could be used as well.

**Figure 8:**
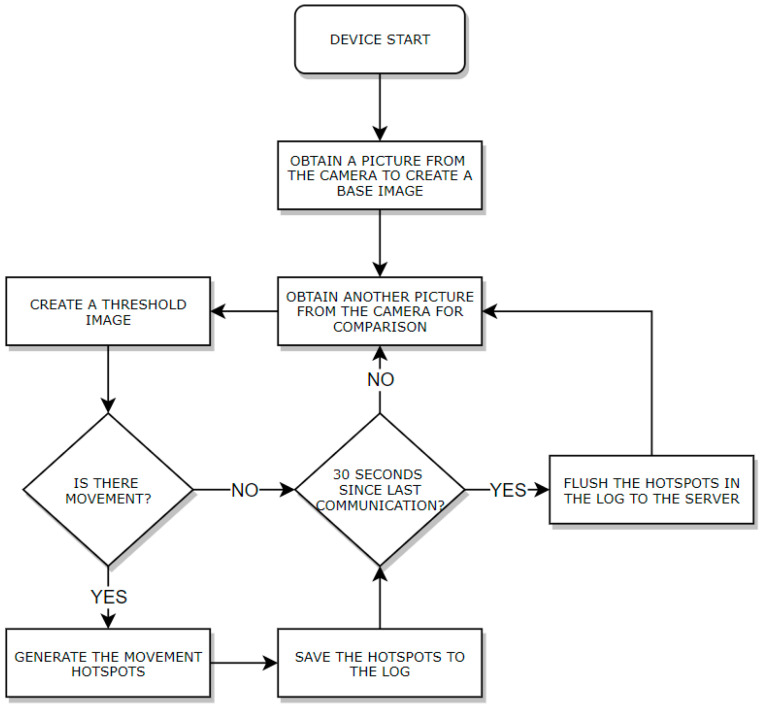
Client operation algorithm.

Figure 8 describes the algorithm implemented by the Monitoring Agent to collect and compare images, find hotspots, and send them to the server (explained in the communication subsection). The device starts up and initially acquires an image to use as a base. In the following instant, the device acquires another image, and then creates a threshold for it. It analyzes if there is movement or not and if so, creates a hotspot entry and saves it into the log. Every 30 s the IoT device tries to upload all the hotspots it finds in that period of time.

**Figure 9:**
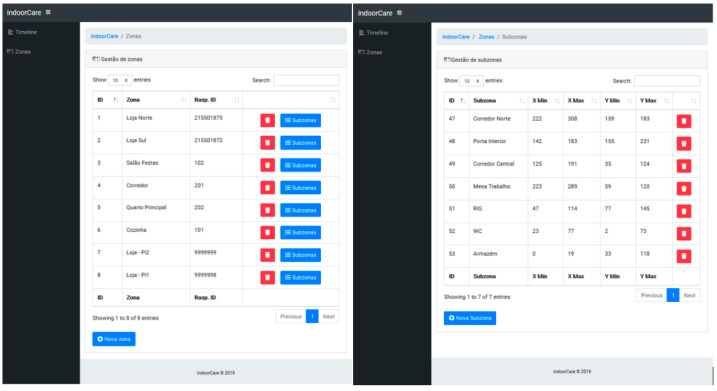
Management of zones and subzones.

On the server side, the Presentation Agent receives and inserts the data into a database, after which it is processed and presented to the user/caregiver. To detect movement in an area, it is necessary to create sub-areas that will work as baselines for comparison with the detected hotspots by the devices. The server prototype provides the management feature to define and manage zones and subzones, as exemplified in Figure 9.

**Figure 10:**
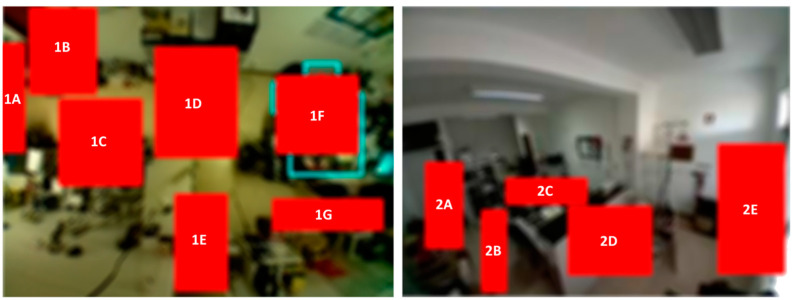
Two zones and several subzones defined for the prototype.

This management feature allows the administrator/system installer to create the zones and the respective subzones that the caregiver want to supervise. It should be noted that to be able to acquire an image of the IoT device used as a monitoring module, a physical action on the equipment is required, namely the junction of two GPIO pins to activate the device’s configuration mode. In Figure 10 are displayed two zones used in the system’s prototype and its subzones, each one identified by an ID. Any hotspot detected within one of the delimited areas corresponds to movement in that subzone.

**Figure 11:**
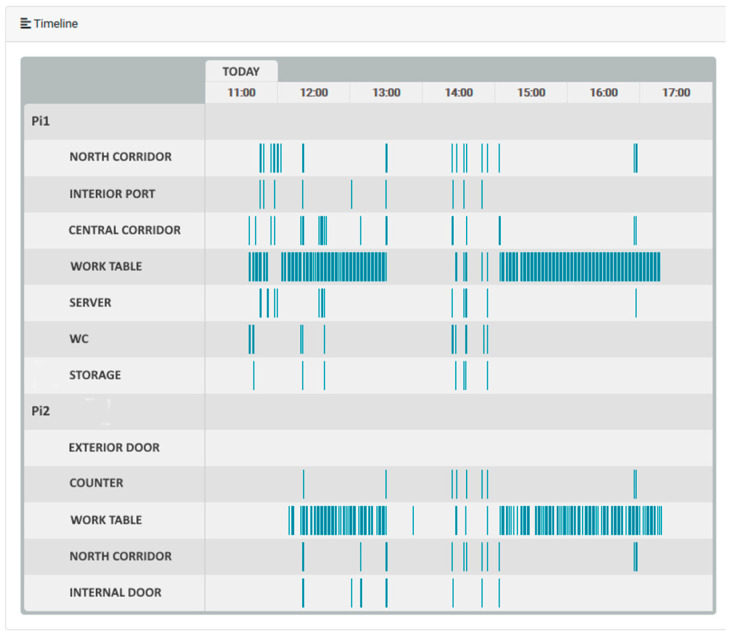
Event timeline.

Following the hotspot detection, this information must be transmitted to the caregiver through a simple and effective interface, mainly because if the interface is too complex, the caregiver may not feel comfortable using it. An example of a simplistic visual interface is the timeline feed of events shown in Figure 11, which is a summary of the events that occurred each day at a certain time. The timeline provides a perception feed of the activity in the elderly home spaces under monitoring, by combining the feeds from the zones into a unique feed, formatted as a timeline grid. For the human caregiver/user, it is very simple and effective to check for specific events [49] and general activity in the house. In the timeline, each blue vertical stripe represents a hotspot detection in that respective subzone, signaling that movement was detected at that time in that area.

With the timeline display, the caregiver can follow the daily life of the elderly and check his routine in a simple and non-intrusive way.

### 4.3. Communication

To be able to send the “converted images” transformed into data to the server, it is necessary for the client to be able to structure this information in such a way that it will be well interpreted at the destination. XML (Extensible Markup Language) [50] is a markup language that allows structuring information in a simple and easily readable way by human beings. It is one of the standards used in the communication of information between information systems and has great flexibility, allowing the creation of the most varied message structures. Another format also widely used in information communication is JSON (JavaScript Object Notation) [50], a compact message format that has less overhead than XML and has been also widely implemented in the industry.

In this prototype, we chose to use XML only because it allows easier reading of messages and facilitates the query of logs, but JSON could also be used. The messages sent in XML from the IoT devices to the server have the following fields:

- datetime: date and time of registration of hotspots, will be grouped in intervals of 30 s for better organization on the server.
- loggerid: the unique identifier of the IoT device that is collecting the information.
- framewidth: the original width of the image that generated the hotspot.
- frameheight: the original height of the image that generated the hotspot.
- matrixwidth: the scale of the matrix width used in this device (to normalize the different resolutions of different cameras).
- matrixheight: the height scale of the matrix used in this device (to normalize the different resolutions of different cameras).
- hotspot: with the x and y coordinates of a hotspot detected at that moment, there may be several at the same moment.

**Figure 12:**
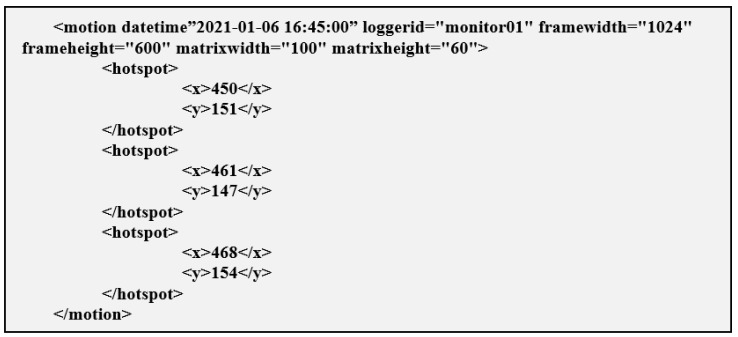
Example of an XML message used.

Figure 12 presents an example of one of these messages, sent periodically to the server.

Client devices calculate hotspots and store this information in a log to be sent every 30 s. In case of communication failure, the Monitoring Agents themselves save the information that was not successfully sent to the server in the log and in the next iteration they try to resend all the pending information.

Initially, it was decided to encrypt the data using symmetric encryption on the clients, but this required unnecessary processing by the IoT devices, so the solution was to delegate this task to the gateway, which would be responsible for creating the VPN bridge with the server and ensure information security and confidentiality.

### 4.4. Movement Data History

One of the advantages of the way the system is designed and implemented is that there is a history of every hotspot detected, and so the processing of movement in the subzones is carried out in the server, and new subzones can be added or rearranged long after the system’s first initialization.

**Figure 13:**
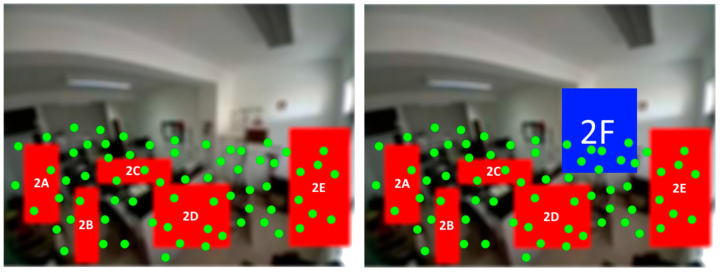
Hotspot’s history.

In Figure 13 is presented an example of how this works. On the left side there are five subzones defined and all the hotspots registered since the system startup; on the right side there is a new subzone defined (2F) after the system initialization. Because a hotspot history exists for each zone, every movement that occurred in that subzone, even before its creation, can be fully visualized in the timeline.

Doing this allows the visualization of all the movement in the new/rearranged subzones, which were not contemplated in the system before, because all the data history related to the detected movement of the entire zone is saved.

## 5. Tests and Optimizations

Due to the current COVID-19 pandemic, it was not possible to carry out tests in real situations with the elderly. All tests performed were simulated in the same house division/area, with specific tests focused on the correct functioning of each module. During the tests, some optimizations were made, namely in the agent present in the IoT devices.

### 5.1. Client Testing

The tests performed on the IoT devices consisted of analyzing the code of the agent developed in python and its ability to perform the necessary operations, namely:

- Acquire images from a camera connected to the IoT device;
- Process the image using OpenCV for motion detection;
- Creation of hotspots for later upload to the server;
- Sending collected hotspots to the server.

**Figure 14:**
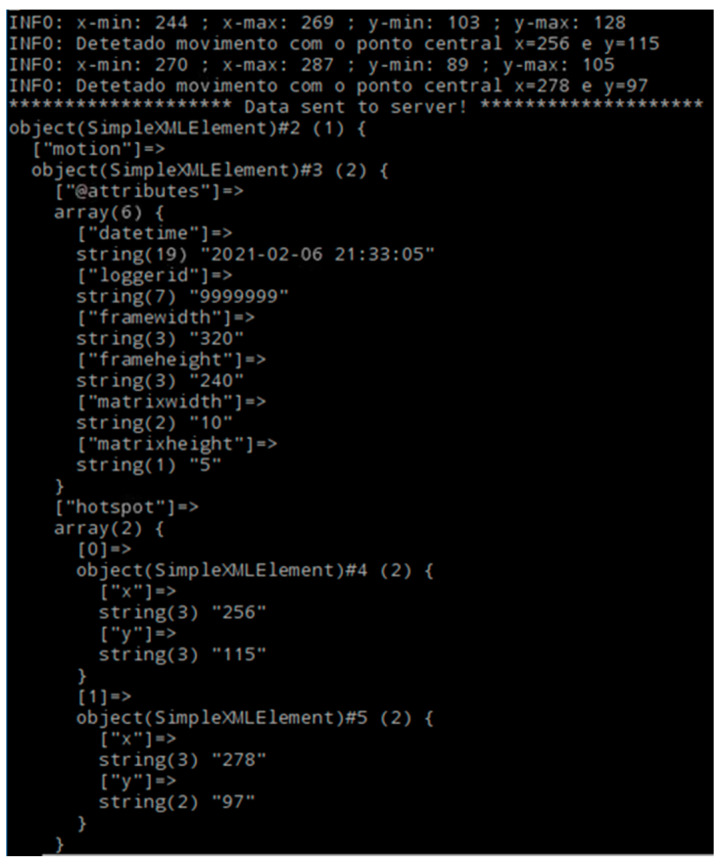
Monitoring Agent output in debug mode.

In Figure 14 is shown the output of the Monitoring Agent, while in debug mode, displaying the image coordinates, where the movement was detected, and the XML message generated to be sent to the server.

**Figure 15:**
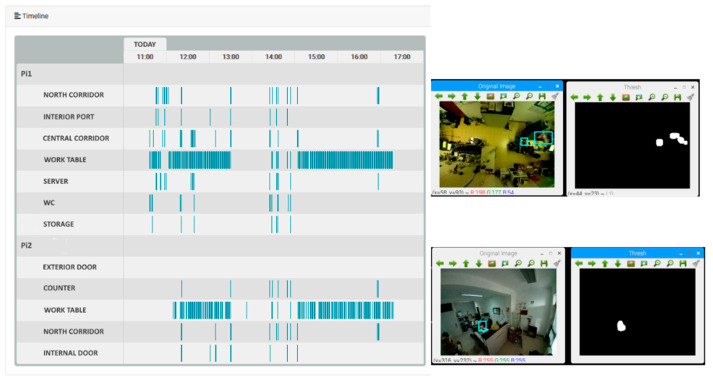
Timeline of events and the respective devices.

Another test involving the IoT devices was to verify if the hotspots generated by the devices were accurate or not; that is, if the motion detected by the devices was consistent with the motion points that appeared on the server’s timeline. In Figure 15 is shown the timeline of the event stream, as displayed by the server, showing movement detection, while on the right side of the figure are shown the outputs of the equipment and the zones they are monitoring.

The tests performed on the system confirmed that the points collected by the device and the movements present in the timeline matched.

### 5.2. Server Testing

The tests carried out on the server focused on the reception and processing of data from the servers and their presentation to the user/caregiver, including:

- Reception of the hotspot in the IoT devices;
- Hotspot data processing and timeline generation;
- Creation and editing of zones and subzones.

**Figure 16:**
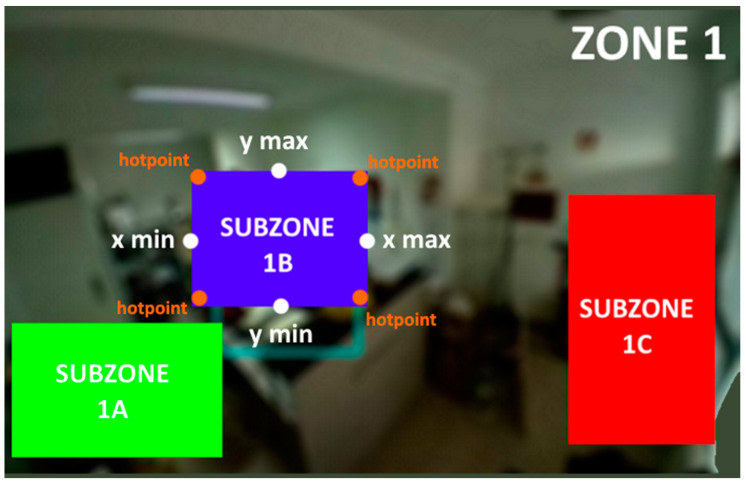
Subzone creation testing.

In Figure 16 is shown an example of how the subzone creation tool of the server was tested; this was accomplished by creating subzones with specific x and y limits and by sending static hotspots generated manually on the IoT device with the corners of the subzone, to verify that the server was placing the hotspot point in the correct pixels on the image and thus generating movement correctly in that subzone.

This test, in particular, served to verify if there was any deviation in the hotspot calculation due to the scale applied to the different image sizes of different types of cameras. Different image capture resolutions were used to see if the same movement coincided in the same subzone, a result that was confirmed at the end.

### 5.3. Timeline Interpretation

The timeline event feed is a key element of the system, for which were conducted some tests to verify if an ordinary person (after very brief training) could understand the information, as presented, and perceive the events that might have generated those data. Due to the current pandemic situation, the tests were executed with only five persons simulating caregivers.

**Figure 17:**
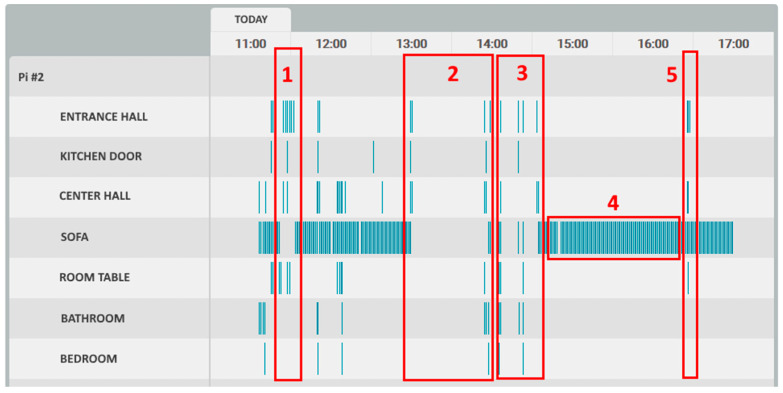
Timeline interpretation.

A test protocol was designed under which the subjects received a hypothetical timeline of the event feed of a day in a hypothetical house. While visualizing the timeline, the subjects were questioned about what they perceived had happened in the house during the day. In Figure 17 is shown a timeline, created for testing purposes only, in which specific numbered points correspond to specific events.

The events were then presented, but not numbered, and the subjects had to match the event with the event number on the timeline. These events were as follows:

- “*Mr. João spent the morning watching television on the sofa and then went to lunch.*”;
- “*Mr. João went to drink water in the kitchen.*”;
- “*Mr. João went to the bathroom.*”;
- “*Mr. João was watching TV for almost 2 h.*”;
- “*Someone knocked on the door and Mr. João went to see who it was.*”.

The results, with a test group of five individuals, are quite positive, with all the individuals confirming that they were able to perceive what happened by reading the timeline. The only exceptions were events 1 and 5, which are very similar in the timeline, and two of the five individuals misinterpreted these two.

### 5.4. Hotspot Detection Optimization

**Figure 18:**
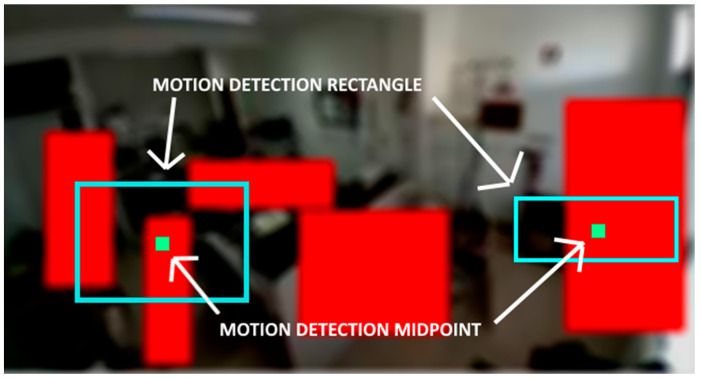
Movement detection optimization.

During the tests on IoT devices, it was noticed that when using an outline rectangle for the movement detection, as the example in Figure 18 shows, the calculation of hotspots sometimes covered two or more subzones, leading to quite a few false positives in subzones where the movement was not happening.

To reduce the number of false positives, it was decided to create a central point in the motion detection rectangle that would represent the midpoint of all the movement that occurred in that specific area of the image. By performing this optimization, and after several tests, it was concluded that when using this midpoint technique, the number of false positives decreased significantly, creating a timeline with much less scattered movement points.

Another advantage of this optimization was the significant decrease (about 50%) in the network traffic to send a hotspot data message, mainly because the messages are in XML and the overhead becomes much smaller, when, in this case, only a pair of X + Y coordinates are transmitted per movement, instead of the two pairs of coordinates to send the rectangle.

### 5.5. Automatic Background Adaptation

The analysis of whether there is movement or not is performed by comparing an image with a previous image (base image), in order to verify if there are differences between them. There is a problem when the base image no longer corresponds to the actual scenario, and small changes were introduced due to non-motion pixel changes. These changes, although not caused by motion, were detected as motion because the pixels in the image changed since the base image.

**Figure 19:**
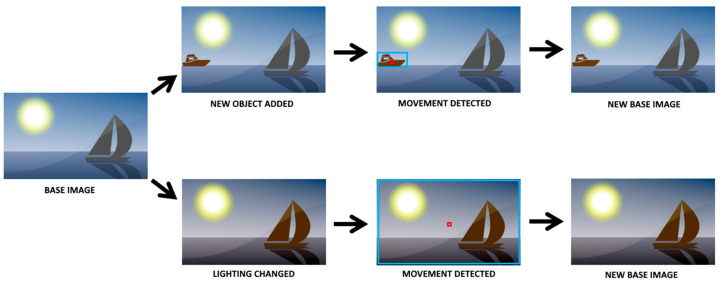
Creation of the new base image.

One of the identified problems was the constant change in the environment that the client was analyzing, either due to the presence of new objects or changes in lighting scenario, such as a light being turned on. To address this problem, in the IoT device was implemented a compensation algorithm that modified the baseline image when required. In Figure 19, two of the problems encountered while testing the system are presented. The first one is when an object enters the background scenario, and it does not exist in the base image. The second is when the scenario lighting changes, and all the pixels change, creating movement in the entire image.

After several tests, an algorithm was created based on the work of [51], to optimize movement detection, that changed the baseline image based on two conditions:

- When no significant movement is detected for more than X seconds (X is variable), which allows the baseline image to be updated to ambient lighting throughout the day.
- When significant movement is detected for more than Y seconds (Y is variable), which happens in at least three different cases: when there is a sudden change in the ambient lighting, when new objects are introduced in the scenario, and when there is real movement in the image.

Regarding the second assumption, when a new base image is created, and if the movement detection continues, then it is because there is real movement detected. In case of a change in lighting or a new object in the scene, after the new base image is created the movement stops.

### 5.6. Cameras with Fisheye Lens

**Figure 20:**
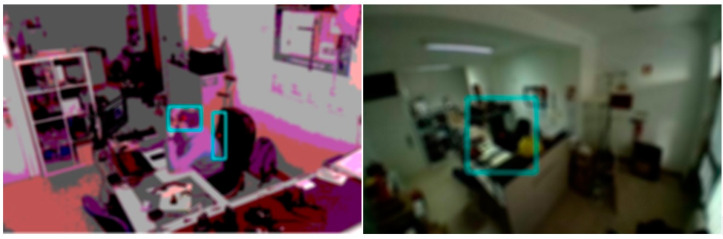
Angle viewing of lens: 72° (**Left**) vs. 160° (**Right**).

During the initial development of the system, cameras with regular lenses were used, with an about 72° viewing angle, which greatly limited the area to be monitored, especially if viewed from above (ceiling of the room). Later, fisheye lenses, with an approximately 160° viewing angle, were installed on the IoT devices, allowing a much larger monitoring area. In Figure 20, there is a lens comparison with normal lenses (72°) on the left and fisheye lenses (160°) on the right.

The equipment is positioned exactly in the same place and the only difference is the camera lens. On the right side, the amount of area is much bigger, which allows a better use of the image for motion detection. With this type of lens, it is also possible to place the equipment on the ceiling of the room and monitor the entire area, as seen in Figure 8.

With this optimization, and after several tests, the fisheye lens installed in the ceiling proved to be the ideal place to position the IoT device and monitor the room. This setup also produced fewer false positives in the movement detection in the subzones, mainly because the line of sight between the camera and the subzone is less likely to be obstructed.

### 5.7. Movement Detection Performance

**Figure 21:**
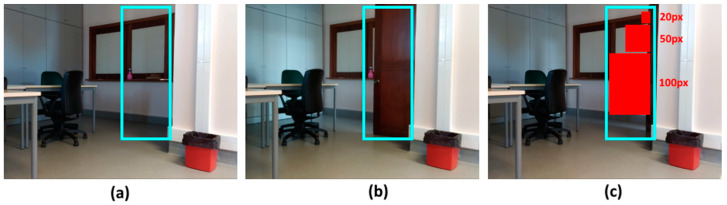
Movement detection of a closed (**a**) and open door (**b**) and the used movement area sizes (**c**).

To test the system’s performance in real motion detection, various sizes of minimum motion area were tested to check how this would influence the system’s execution. Figure 21 shows the test scenario with a defined sub-area (a) to detect the movement of the door (b). Three sizes of minimum area were used to detect the same movement of the door (c), which were 20 px, 50 px, and 100 px. These sizes were defined in particular for this test and another set could be defined; the purpose was only to check whether the minimum detection area influenced the detection of the same movement.

**Figure 22:**
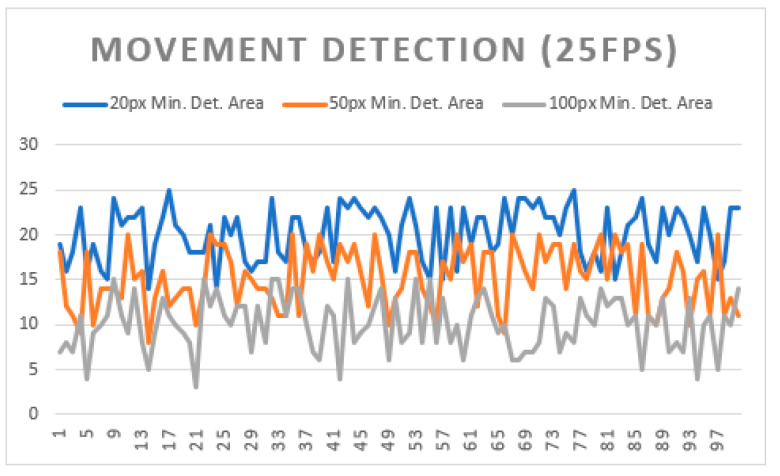
Movement detection with multiple minimum area sizes.

The graph in Figure 22 shows the results obtained from the tests carried out. As can be seen, when using a smaller motion detection area, the system can recognize the same motion/movement in that subzone more often than when the area is larger.

This result is expected and is reflected in previous tests, since the detection of the changed pixels is sometimes very fragmented, creating several small detection areas that are discarded if the minimum size of these to be considered valid is too high.

## 6. Conclusions and Future Work

In this work, an effective and low-cost indoor monitoring system was proposed to help caregivers take care of the elderly by monitoring their daily lives from a distance. This system brings the advantages of knowing where the elderly person is and the activity dynamics in the house, while fully respecting the elderly person’s privacy, thus creating a daily movement record of the elderly person.

The test results of the prototype show that it is possible to use low-price and low-performance IoT equipment, namely a Raspberry Pi Zero W, to build a system that performs monitoring in a specific zone in the house and its associated subzones. In addition, the tests also indicate that the usage of the timeline event feed model is very effective to display the activity inside a home and that it is very simple to interact with.

As future work, an optimization that can be made in terms of processing on the Raspberry Pi is the implementation of gray areas, i.e., areas that will never have points of interest for motion detection. The detections that happen in these subzones (e.g., reflections) are not sent to the server, leading to less false positives and a smaller amount of information to be sent to the server. Other improvements in this type of system include the implementation of automatic alerts, which could be of two types: a “Non-movement alert”, which would inform the caregiver when something abnormal happens in the elderly’s routine; or a “Too long alert”, which would be used to inform the caregiver that, after movement was detected in an specific area, it suddenly stopped, informing the caregiver that something may be happening.

## Figures and Tables

**Table 1:** Comparison between the solutions presented in the related work section.

Reference	Case Study	Why Was Chosen
[23]	Hand gesture detection	Image data extraction using OpenCV
[24]	Video processing on Raspberry PI	OpenCV on Raspberry PI
[25]	Human facial recognition	OpenCV on Raspberry PI
[27]	Extraction of vehicle information	OpenCV on Raspberry PI
[28]	Surveillance system	Image acquisition on Raspberry PI
[29]	Indoor/outdoor person detection	Uses RFID tags to detect humans
[30]	Indoor human detection	Uses infrared sensor to detect humans
[31]	Indoor monitoring	Uses IoT devices with AI
IndoorCare	Indoor human monitoring	Uses IoT devices with Computer Vision

## Data Availability

Not applicable.
